# Insights Into the Pathogenesis of Bullous Pemphigoid: The Role of Complement-Independent Mechanisms

**DOI:** 10.3389/fimmu.2022.912876

**Published:** 2022-07-07

**Authors:** Connor Cole, Keshavamurthy Vinay, Luca Borradori, Kyle T. Amber

**Affiliations:** ^1^ Division of Dermatology, Rush University Medical Center, Chicago, IL, United States; ^2^ Department of Dermatology, Venereology and Leprology, Postgraduate Institute of Medical Education and Research, Chandigarh, India; ^3^ Department of Dermatology, University Hospital Inselspital Bern, University of Bern, Bern, Switzerland; ^4^ Department of Internal Medicine, Rush University Medical Center, Chicago, IL, United States

**Keywords:** bullous pemphigoid, complement - immunological terms, autoimmune blistering diseases, eosinophils – immunology, igE (Immunoglobulin E), BP180, BP230

## Abstract

Bullous pemphigoid is an autoimmune blistering disease caused by autoantibodies targeting BP180 and BP230. While deposits of IgG and/or complement along the epidermal basement membrane are typically seen suggesting complement -mediated pathogenesis, several recent lines of evidence point towards complement-independent pathways contributing to tissue damage and subepidermal blister formation. Notable pathways include macropinocytosis of IgG-BP180 complexes resulting in depletion of cellular BP180, direct induction of pro-inflammatory cytokines from keratinocytes, as well as IgE autoantibody- and eosinophil-mediated effects. We review these mechanisms which open new perspectives on novel targeted treatment modalities.

## Introduction

Bullous pemphigoid (BP) is the most frequent autoimmune subepidermal blistering disease associated with an autoantibody response directed against the BP antigen 180 (BP180, BPAG2 or type XVII collagen) and the BP antigen 230 (BP230 or BPAG1-e). The latter are components of junctional adhesion complexes called hemidesmosomes that promote dermo-epidermal cohesion (1). Characteristically, BP is an intensely pruritic eruption with generalized blistering. However, in early stages or in atypical variants of the disease, only localized or generalized excoriated, eczematous, or urticarial lesions may be present. The disease, which has a chronic course, typically affects the older population after the age of 65 and has a significant impact on both the quality of life and life-expectancy (2). The one-year mortality ranges from 13% to 40%, while the mortality rate of patients with BP seems to be at least three times higher than that of age- and sex-matched subjects (3). The annual incidence has been estimated to be at least 6–13 new cases per million population with a striking increase after the age of 80 years (with more than 300 cases per million in individuals). Nonetheless, in the last two decades, there is evidence indicating a two to four-fold rise of the overall incidence of BP in the population, most likely due to the better recognition of atypical forms of BP and the increasing relative size of older age groups (4).

A recent consensus guideline on management of BP primarily recommends the use of high potency topical steroids and systemic corticosteroids as first-line therapeutic options (5). Immunomodulatory and immunosuppressive drugs may be considered in treatment-resistant cases or in cases at increased risk for steroid-related adverse events or in the presence of contraindications to systemic steroids. In recent years, a number of biologics have been used with promising results, such as omalizumab, dupilumab, interleukin-17, and IL-5Rα inhibitors (6, 7). In addition, a recently published phase 2a trial examined the use of nomacopan, a leukotriene B4 and C5 inhibitor, in BP patients. The drug appears to be well-tolerated by patients and has therapeutic potential for reducing acute BP flares (8). As BP is more common in the elderly, balancing management with patient comorbidities is almost invariably challenging. The efficacy of current treatments is limited and relatively unsatisfactory; patients’ unmet needs remain significant. Hopefully, several ongoing trials will allow more effective and better tolerated therapies to be validated in the near future. Such therapies should facilitate and improve the overall management of affected patients, which primarily consist of fragile and debilitated individuals.

## Pathogenesis of Bullous Pemphigoid

There is ample evidence indicating that BP occurs due to a loss of immune tolerance leading to autoantibody formation against BP180 and BP230. BP180 is transmembrane protein with a large collagenous extra-cellular domain serving as an adhesion molecule. Its ectodomain binds to laminin 332 and type IV collagen, connecting the basal keratinocytes to the extracellular matrix of the epidermal basement membrane (9–11). BP230, the epithelial isoform of BPAG1, is a cytoplasmic protein of the plakin family of cytolinkers. It primarily connects the keratin intermediate filament system to hemidesmosomes at the basal keratinocyte cell membrane (1, 9, 12). Patients’ sera recognize multiple antigenic regions on both target antigens, although the NC16A domain, on the extracellular membrane of BP180, contains the immunodominant antigenic determinants (13, 14). The autoreactive B and T cell response in BP is primarily directed at this region of BP180 (15, 16). BP autoantibodies lead to an inflammatory response with a large number of eosinophils and, to a lesser degree, neutrophils, migrating to the dermis and degranulating. These cells contain and release upon activation dozens of cytokines, chemokines, hydrolytic degrading enzymes, including matrix metalloprotease 9 (MMP9) and neutrophil elastase, as well reactive oxygen species. This inflammatory cascade ultimately leads to tissue damage and subepidermal blister formation (17–21).

*In vitro* and *in vivo* studies have allowed the characterization of several pathways critically involved in BP pathogenesis that directly contribute to tissue damage. Among these, activation of the complement system with production of anaphylatoxins, and activation of the innate immune response with subsequent recruitment and activation of basophils, eosinophils, neutrophils, monocytes/macrophages, and mast cells, play a key role in BP (15, 19, 22–27). Complement components, including C1q, C3, C4, and the membrane attack complex (MAC) are usually found detected along the dermal-epidermal junction (DEJ) in the skin of both patients with BP and of mice with experimentally induced BP (28–31). Furthermore, complement proteins including anaphylatoxins are detectable in the blister fluid of BP patients (32). The presence of tissue-bound complement components and/or the ability of circulating autoantibodies to mediate complement activation are also likely to affect clinical and histopathological features, including overall disease activity, in affected patients (33). For example, the presence of tissue-bound C3 in the skin of BP positively correlates with the presence of circulating anti-BP180 antibodies targeting the NC16A domain (34). While the importance of complement was described as early as the 1970s (35), increasing evidence has emerged pointing to the presence of complement-independent pathways in mediating tissue damage and subepidermal formation in BP.

This review will seek to summarize the current understanding of complement-independent mechanisms in BP and provide a reference framework for future research aimed at further elucidating these processes. This new knowledge is expected to facilitate the development of new treatment modalities that should benefit the management of BP patients in the near future.

## Complement-Independent Pathways in Tissue Damage and in Dermo-Epidermal Disadhesion

In the last decade, a number of laboratories have provided convincing evidence that complement-independent processes are implicated in the pathogenesis of BP, directly contributing to inflammation, tissue damage and dermo-epidermal separation. This idea is substantiated by a number of *in vitro* and *in vivo* experiments as well as clinical observations, including:

**1)** Serum derived anti-NC16A IgG antibodies and recombinant anti human NC16a IgG antibodies impair keratinocyte adhesion and deplete BP180 by induction of macropinocytosis (36–39); **2)** Passive transfer of F(ab’)_2_ fragments of the human BP or IgG antibodies, against BP180, that cannot activate complement, are able to cause skin fragility in neonatal BP180-humanized mice (36); **3)** C5^-/-^ mice as well as C5ar1^-/-^ mice injected with anti-NC15A antibodies, the murine analog to NC16A in humans, develop a BP-like phenotype, although its severity is milder when compared to that of wild type (WT) mice (40). In the latter study, pharmacologic inhibition of C5a receptor 1 fails to reduce clinical disease or neutrophil infiltration in mice with established cutaneous disease (40); Notably, this study demonstrates that inhibition of complement has therapeutic benefit, once again reaffirming the importance of complement in BP. However, C5ar^-/-^ mice demonstrated a relatively increased extent of skin lesions following BP-IgG injection, raising the possibility of complement-independent induced blistering as well **4)** Production of neutrophil reactive oxygen species (ROS), which contribute to tissue damage, does not differ between WT, C5ar1^-/-^, and C5ar2^-/-^ mice (40), **5)** Passive transfer of human BP-IgG into C3^-/-^ BP180 humanized mice develop blisters (39); **6)** Non-complement binding autoantibodies are able to cause blister formation *in vivo* (39, 41); **7)** BP-IgG antibodies are able to induce IL-6, IL-8, and Hsp90 expression from cultured keratinocytes independent of complement (42–48); **8)** IgG4 autoantibodies, which are the dominant IgG isotype in over 50% of BP patients, are able to induce blistering in cryosection assays (41, 49–54) **9)** IgE autoantibodies and eosinophils contribute to blister formation by means of various mechanisms including secretion of proteases, eosinophil degranulation, and extracellular traps, as well as cytokine and chemokine release in a complement independent manner. However, IgE antibodies very rarely form in isolation of IgG. As such, while they are definitively pathogenic, their contribution relative to IgG is unclear. Finally, **10**) in up to 20% of biopsy specimens obtained from BP patients, there is no evidence for complement deposition as assessed by direct immunofluorescence microscopy (DIF) (55). However, this study was limited by use of a single detection antibody against complement, specifically the C3c component. Sensitivity may increase with the use of multiple antibodies. Likewise, a case report of a patient developing BP despite C4 deficiency provides further support (56)

The recent Phase 2a trial results of the complement inhibiting drug nomacopan offer an important caveat to the prospect of physiologically significant complement-independent effects (8). In this study, 7 of 9 patients saw significant reductions in skin severity index scores by six weeks. However, the subjects who were non-responders to such complement inhibition raises the possibility of tissue damage and blister induction occurring outside of the complement system in these patients. Furthermore, randomized trials with much larger sample sizes are needed to truly assess the therapeutic efficacy of this drug.

## Non-Immunologic Induction of Blistering by Anti-BP180 Antibodies on Keratinocytes

The direct non-immunologic, but complex biologic impact of anti-BP180 antibodies on basal keratinocytes represents an important means by which complement-independent mechanisms contribute to basement membrane disadhesion and dermo-epidermal blistering. Both ubiquitin- and proteasome-mediated degradation of BP180, as well as macropinocytic internalization of BP180 appear to be involved (57). Notably, this is seen with antibodies targeting the NC16a domain of BP180, but not the c-terminus, suggesting epitope dependent pathogenicity (58).

Early studies by Kitajima et al. first showed that the binding of anti-BP180 antibodies results in internalization of BP180 in cultured epidermal cells (59, 60). Further work by Iwata et al. confirmed that incubation of anti-BP-IgG autoantibodies causes internalization of BP180 from the cell membrane and provided evidence indicating that BP180 is depleted from the keratinocyte. In these experiments, BP-IgG treatment was able to reduce the amount of BP180 from cells by roughly 40% and 85% after two and six hours of respective incubation as assessed by immunoblotting. Notably, while BP180 was decreased, the amount of the α6β4 integrin, a key component of hemidesmosomes, remained unchanged, indicating that the effect on BP180 was specific. By semiquantitative analysis of BP180 content in BP patients’ skin by immunoblotting, the authors also found a reduction of ~40% of BP180 in BP patients’ skin when compared to that of control subjects. Finally, in vibration detaching assay using cultured keratinocytes, BP-IgG treatment caused a significant reduction of the adhesion of cells to the culture plate (61). Another study showed that both BP-IgG and BP-IgE are capable of directly binding to the surface of cultured human keratinocytes with subsequent loss of hemidesmosomes at the basement membrane zone (BMZ) (44). The studies mentioned above used *in vitro* models exclusively, and generalizations regarding this apparent BP180 internalization and subsequent loss of adhesion to living systems are limited.

In 2012, Natsuga et al. reported that rabbit antibodies raised against a distinct portion of the human NC16A region of BP180 (Arg^522^ to Gln^545^) decrease BP180 expression in cultured NHKs (36). More strikingly, the same group also found that the injection of the F(ab’)_2_ fragments of the rabbit anti-BP180-NC16A antibodies, thus lacking the complement-binding Fc domain, was able to cause dermal-epidermal splitting in neonatal BP180-humanized mice and also decreased expression of BP180 in murine skin by immunoblotting. Nevertheless, the observation that not all mice injected with the F(ab’)_2_ fragments displayed skin fragility implies that the anti-NC16A F(ab’)_2_ fragments have a less potent effect than BP-IgG (36). It is important to note that this study utilized neonatal mice. Skin fragility and neonatal immune response may not be predictive of human responses. Hiroyasu et al. subsequently confirmed the findings of Natsuga et al. using cultured 804G cells and normal human epidermal keratinocytes (NHEKs) as well as BP-IgG(Fab’)2 and BP-IgG Fab fragments (37). Hence, these observations strongly indicate that skin detachment in BP not only depends on a complement-mediated inflammatory cascade, but also involves a direct effect of BP antibodies on the adhesive function and cell expression of BP180, which directly impairs dermo-epidermal cohesion. It is unclear why previous mouse model studies failed to observe a direct effect of IgG(Fab’)2 fragments against BP180 on keratinocyte adhesions. These apparent discrepancies may be related to the variable experimental conditions of the used *in vitro* assays and *in vivo* models (22, 62, 63).

It has been speculated that blistering in BP first requires a weakening of the adhesive strength of keratinocytes, which is then accompanied by an inflammatory response that ultimately causes dermo-epidermal separation (37, 61). This model, which is not yet substantiated by experimental data, can however be applied on a subset of patients with pauci-inflammatory BP and other subepidermal bullous autoimmune diseases in which blistering primarily results from mechanical trauma and friction. This phenomenon is typically observed in patients with the mechano-bullous form of epidermolysis bullosa acquisita, with antibodies directed against type VII collagen.

BP-IgG induced internalization of BP180 occurs through the macropinocytosis pathway. In fluid uptake assays, the addition of macropinocytosis inhibitors are able to block the internalization of BP180 in both cultured 804G cells and NHEKs. This process seems to occur following a calcium-dependent phosphorylation of the intracellular domain of BP180 by the protein kinase C pathway (38). Inhibition of the macropinocytosis pathway is also able to block the negative effect of BP-IgG on the adhesive strength of cultured NHEKs following treatment (37). The entire BP180 molecule seems to be internalized as a complex bound with BP-IgG. The impact of this internalization of BP180 on hemidesmosome formation *in vitro* and potentially *in vivo* remains to be assessed (64).

Ujiie et al. also characterized the mechanisms by which BP-IgG induced BP180 depletion takes place (39), confirming that anti-BP180 BP-IgG can induce blistering in complement-deficient mice. Notably, these researchers have utilized a monoclonal antibody, mIgG2c (TS4-2) which has high complement activation activity, low ability to deplete BP180, and low affinity to hNC16A. The passive transfer of this mIgG2c anti-BP180 monoclonal antibody failed to cause blistering in most mice. Ujiie et al. also showed that BP180 is ubiquinated following treatment with BP-IgG. In fact, addition of a proteasome inhibitor, MG-132, prevents the depletion of BP180 in a dose-dependent manner. These results thus indicate that the ubiquitin/proteasome pathway is implicated in BP180 depletion. Noteworthy, in this latter experimental model, mice injected with the monoclonal antibody rhIgG4 against the human NC16A domain of BP180 (but unable to activate the complement) still developed blistering despite the administration of a proteasome inhibitor. This observation indicates that internalization of BP180 and adhesive weakening most likely precede the degradation of BP180 *via* proteasomes. In this context, it should be mentioned that proteins that enter cells *via* macropinocytosis are usually degraded by lysosomes. However, proteasomes may also be involved under certain circumstances, such as in relation to cross-presentation of antigens on the major histocompatibility complex I pathway (65). Hence, it remains unclear if BP180 is only degraded by proteasomal pathways or if lysosomes and/or other processes are also implicated (9).

## Regulation of Inflammatory Responses by BP180 in Keratinocytes

In addition to the direct non-inflammatory effects of anti-BP180 autoantibodies, there is growing evidence for complement-independent inflammatory mechanisms by which BP antibodies can induce disease. Specifically, keratinocytes are able to secrete a variety of proinflammatory cytokines which appear to be pathogenetically relevant. Specifically, in 2000 Schmidt et al. found that treatment of cultured NHEKs with anti-NC16A BP-IgG, and not control IgG, results in an increased expression of IL-6 and IL-8 in a time- and concentration-dependent manner while IL-1α, IL-1-β, IL-10, and TNF-α were not detected and MCP-1 levels remained unchanged (46). Molecules such as IL-1β and TNF-α are known to upregulate complement factors (66). The upregulation of IL-6 and IL-8 was detected at both the mRNA and protein level. In addition, blocking the immunoreactivity of two distinct epitopes, NC16A and NC16A2, prevented the upsurge in IL-8. Also of note, since BP-IgG treatment of BP180-deficient keratinocytes did not cause an increased release of IL-6 or IL-8, these results suggest that the specific interaction between BP autoantibodies and BP180 ectodomain initiates an intracellular signal transduction pathway affecting transcription and translation with an increased release of keratinocyte-derived IL-6 and IL-8 (46).

Messingham and colleagues expanded on these results finding that not only IgG but also IgE anti-BP antibodies are capable of stimulating IL-6 and IL-8 production in cultured human keratinocytes and organ culture (44). Similar results were obtained following treatment with F(ab’)_2_ and Fab fragments prepared from IgE and IgG, confirming that these effects occur in an FcR-independent manner. Noteworthy, IgE appears to be a more potent stimulator of cytokine production compared to IgG (44). In this context, we recently identified a similar pro-inflammatory response from keratinocytes treated with IgG obtained from patients with laminin-332 pemphigoid, particularly with autoantibodies against the β3 subunit (67).

**Figure 1:**
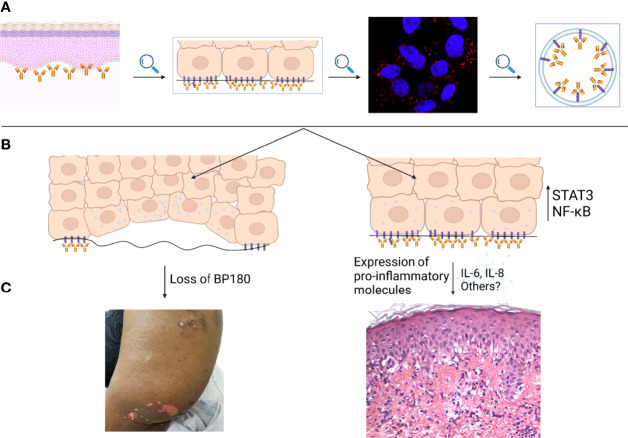
Keratinocyte mediated, complement-independent pathways in bullous pemphigoid. **(A)** IgG autoantibodies targeting BP180 bind to the epidermal basement membrane. IgG-BP180 complexes undergo macropinocytosis as shown by immunostain for EEA-1. **(B)** Internalized IgG-BP180 complexes lead to both loss of cellular BP180 and increased expression of proinflammatory mediators such as IL-6 and IL-8, resulting in both **(C)** pauci-inflammatory and pro-inflammatory complement-independent mechanisms.

IL-8 derived from keratinocytes is chemotactic for neutrophils (68). The latter are critical for the formation of blisters in both animal and *in vitro* BP models (23, 69). Furthermore, Liu et al. showed that intradermal IL-8 injection into C-5 deficient mice can reverse their resistance to the pathogenic effects of rabbit-derived BP-IgG monoclonal antibodies (23). IL-6 is another pro-inflammatory cytokine. It is secreted by keratinocytes and can penetrate the BMZ (70). Its contribution in tissue damage is attested by the observation that IL-6-depleted mice do not develop blistering in a BP model (23). Finally, IL-6 and IL-8 levels are elevated in both the sera and blister fluid of BP patients (71, 72). Serum levels of these interleukins have also been correlated with disease activity in BP (73). Overall these observations indicate that keratinocytes, by releasing pro-inflammatory cytokines, also play a previously unrecognized role in the pathogenesis of pemphigoid diseases (67). Keratinocyte dependent complement-independent mechanisms are summarized in **Figure 1**.

Pharmacologic studies have also been useful to gain better insight into the inflammatory processes triggered by anti-BP antibodies (42, 45, 47). By ELISA, Tukaj et al. found that calcitriol decreased the BP-IgG-induced release of IL-6 and IL-8 in human keratinocytes in a dose- and time-dependent fashion (42). Moreover, this vitamin D metabolite reduced phosphorylation of STAT3 and suppressed NF-κβ activity in keratinocytes treated with BP-IgG, but did not affect levels of heat shock protein (Hsp) 70 or the vitamin D receptor (VDR), an observation implying that NF-κβ and STAT3 are both involved in mediating the pro-inflammatory effects. Dapsone, an anti-inflammatory agent used in BP management, was found to specifically suppress the release of IL-8, but not of IL-6, from NHEKs treated with BP-IgG antibodies isolated from both human and rabbit sera, in a dose-dependent manner (45). Evidence was further provided suggesting that the effect of dapsone occurs at the post-transcriptional level (45). Notably, dapsone can also exert a significant inhibitory effect on neutrophils (74). Therefore, conclusions drawn on the complement-independent mechanisms of BP from dapsone’s efficacy in the disease may be limited.

Hsp90 has been linked to the synthesis of various cytokines such as TNF-α, IL-1, IL-6, and IL-8 (48, 75–77). In human epidermal keratinocytes treated with BP-IgG, blockade of Hsp90 with 17-DMAG is able to suppress the IL-8, but not IL-6, release in a dose- and time-dependent manner (47). Blocking Hsp90 also impaired the NF-κβ p65 subunit activity in BP-IgG stimulated keratinocytes. These findings suggest that Hsp90 also exerts a regulatory role in BP-IgG-induced production of IL-8.

Van Den Bergh et al. sought to assess whether BP180 is directly involved in modulating this pro-inflammatory response (43). For this purpose, they measured IL-8 response under various inflammatory stimuli in both normal keratinocytes and in BP180-deficient keratinocytes derived from either a junctional epidermolysis bullosa patient or after shRNA-mediated knockdown of BP180. The BP180-deficient keratinocytes showed a dysregulated higher IL-8 response after treatment with lipopolysaccharide (LPS), ultraviolet-B radiation or tumor necrosis factor compared to normal human keratinocytes. Notably, inhibition of NF-κβ, but not p38MAPK, was able to normalize this response. The same group also found that LPS treatment of BP180-deficient keratinocytes increases the expression of an NF-κβ-driven reporter compared to normal cells. In LPS-treated cells, inhibition of NF-κβ activity in BP180-deficient keratinocytes normalized their IL-8 response. The results are in line with the idea that the effects of BP180 on IL-8 response are mediated by NF-κβ. Together, these results point toward BP180 serving as regulator of IL-8 involved inflammatory response of keratinocytes. It is as of yet unclear if autoantibodies to BP180 affect its interactions with other hemidesmosomal components, such as the α6β4 integrin and extracellular proteins, and if disturbance of this network has an impact on the inflammatory response. Importantly, many of the aforementioned studies looking at these inflammatory mechanisms and their regulation are limited by reliance on *in vitro* data. As such, drawing conclusions regarding the *in vivo* response must be done carefully.

In another study (78), genetically engineered mice which expressed a NC16A-truncated BP180 developed spontaneous inflammation of the skin and exhibited severe pruritus, compromised skin barrier, increased serum IgE, and immune cell infiltration. The pruritus was found to be independent of adaptive immunity or histamine, but was related to an increased expression of TSLP. This study suggests that dysfunction or structural alteration of BP180 is sufficient to trigger an inflammatory response similar to that seen in BP patients.

Finally, the effects of IgG4 autoantibodies provide evidence for other complement-independent inflammatory mechanisms in bullous pemphigoid. While IgG1 and IgG3 antibodies are known to fix complement, the IgG4 subclass does not (79). In 2007, Mihai et al. isolated both IgG1 and IgG4 BP antibodies from patient sera and introduced them into an *ex vivo* experimental model. Although the IgG4 antibodies did not activate complement, they were able to induce dermo-epidermal spitting and tissue damage *via* leukocyte recruitment and activation (79). While the pathogenic potential of the IgG4 autoantibodies was significantly less than that of IgG1 in this experiment, the study demonstrates the capability of IgG4 to induce a BP-like phenotype in the absence of complement, and introduces a novel role for IgG4 in this disease.

## Eosinophil and IgE Mediated Blistering

Eosinophils can directly mediate dermo-epidermal separation in the presence of either IgG or IgE (80, 81). In both instances, complement is not required. Eosinophils, which are typically abundantly present in lesional skin of BP patients, play an important role in tissue damage by means of different mechanisms (82–86). Eosinophils are capable of secreting the matrix metalloproteinase 9 (MMP-9), which can degrade BP180 and thus contribute to dermo-epidermal separation, by cleaving the extracellular collagenous domain of BP180 and other proteins (21, 87–90).

Degranulated proteins from eosinophils can be detected in both the serum and blister fluid of BP patients (91–94). Eosinophil granules have also been found along the BMZ in patients with BP (94, 95). Release and deposition of eosinophil granules appear to be present even in the early stages of BP lesions (96, 97). We have demonstrated that the granule proteins eosinophil cationic protein (ECP) and eosinophil derived neurotoxin (EDN) induce keratinocyte expression of IL-5, eotaxin-1, and RANTES, as well as reactive oxygen species formation. ECP but not EDN is able to directly induce keratinocyte detachment (98).

Eosinophils can also produce extracellular traps (EETs) which are made up of granule proteins, DNA, and nuclear components in a network-like structure which can expand to be 15 times larger than the cell itself (99). EETs have been found to be present in BP. Based on *ex vivo* data obtained from experiments involving human skin and isolated eosinophils showing that dermo-epidermal separation is reduced with DNase affecting EETs, the latter may be directly involved in the amplification of the inflammatory response, although the exact mechanisms remain still unknown (100). It should be noted that neutrophil extracellular traps (NETs) may also play a role in BP. Using immunodetection of patient skin biopsies, NET formation has been shown to be associated with BP (101). Additionally, levels of NET biomarkers are correlated to BP disease activity (102). Thus, it is likely that these complexes play a role in the tissue damage involved in this disease. However, given the fact that neutrophils are likely recruited at least in part by complement (103), it is unclear whether these NET-related mechanisms may truly be complement-independent.

As described previously, eosinophils are involved in BP pathogenesis by mediating the effects of anti-BP180 IgE antibodies and contributing to dermo-epidermal separation (81). Anti-BP180 IgE autoantibodies are present in the majority of BP patients, and their levels are correlated with disease activity (104–107). In mice with grafted human skin, injection of anti-NC16A IgE resulted in inflammation with development of erythematous skin lesions and dermo-epidermal separation. Influx and degranulation of eosinophils have been here implicated (108). Similar results were obtained using human cryosection dermis in which IgE injection led to DEJ separation with associated eosinophil infiltration. The activation of eosinophils was mediated through the FCεRI receptor (109–111). Notably, the amount of anti-BP180 IgE and IgG was correlated to levels of circulating eosinophils in BP sera (111). Moreover, IgE autoantibodies against a component of the shed ectodomain of BPAG2 induce pruritus, erythema, eosinophil infiltration, and blistering when passively transferred (112).

Eosinophils also directly contribute to BP symptomatology by producing IL-31, a known pruritogen. Pruritus is a key feature of BP and can be a presenting symptom even in the absence of specific skin lesions (13). IL-31 activates endothelin-1 and causes subsequent upregulation of brain natriuretic peptide (BNP), an important mediator of pruritus (113, 114). IL-31 is known to be produced by eosinophils (107, 115), and increased levels of IL-31 have been found in both the lesional skin and serum of BP patients (116). Recent evidence even suggests that eosinophils are the central source of IL-31 in BP (117). Eosinophil-derived IL-31 certainly plays a role in BP itching, but it is still unclear whether this is the primary mediator of pruritus in BP or if other pathways are paramount.

The most compelling evidence for the role of eosinophils in mediating tissue injury in BP comes from Lin et al. who generated a transgenic mouse which expressed human hNC16A as well as the human FCεRI (118). In these mice, anti-NC16A IgE produced subepidermal splitting along with eosinophil infiltration and deposition of IgE along the epidermal BMZ. BP-IgE-induced blistering required the presence of eosinophils. In this model, the intensity of eosinophil infiltration also correlated with disease severity. Overall, these findings not only support the pathogenicity of anti-NC16A IgE antibodies in BP, but also show that eosinophils are the mediator in this process (118).

**Figure 2:**
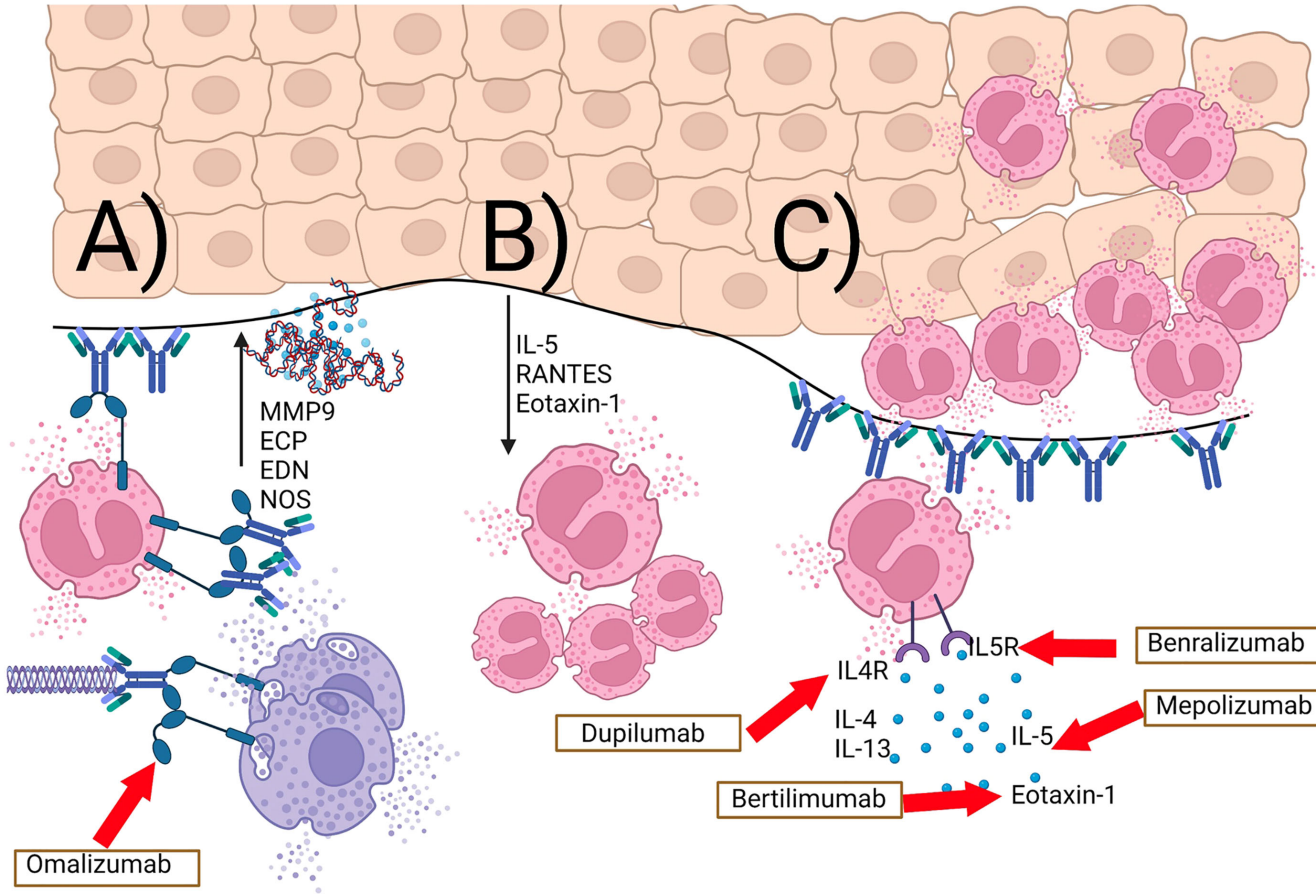
IgE- and eosinophil-mediated complement-independent pathways in bullous pemphigoid. **(A)** BP180 IgE autoantibodies and BP180-IgE complexes bind to the cutaneous basement membrane and the FcϵR1 on eosinophils as well as mast cells and basophils. This results in release of proteases (e.g. MMP9), eosinophil granule proteins (ECP, EDN), eosinophil extracellular traps, as well as reactive nitric oxide-derived oxidants (NOS). **(B)** Keratinocytes release IL-5, RANTES, and eotaxin-1 as a response to eosinophil granule proteins. **(C)** this positive feedback loop results in an increase in tissue eosinophilia and eosinophilic spongiosis. Inhibitory therapeutic antibodies are shown in boxes with red arrows leading to their downstream target.

The work of Freire et al. further characterized the complex role of IgE antibodies in BP (119). Using ELISA and immunofluorescence to examine BP patient sera and skin respectively, they detected increased levels of both anti-BP180 and anti-BP230 IgE compared to healthy controls. The former were found to interact with the same NC16A region of BP180 known to be recognized by IgG. Furthermore, direct immunofluorescence studies showed the majority of BP patients (compared to none of the healthy controls) to have IgE+ cells in their skin. Surprisingly, IgE was rarely detected at the BMZ, and instead was primarily associated with mast cells and eosinophils in the dermis. The study also identified fragments of the extracellular domain of BP180 in the dermis and BMZ, often co-localized with IgE+ cells. These findings indicate that BP180 and IgE can form complexes on the same cells. Further degranulation assays revealed that these IgE-BP180 complexes are capable of cross-linking FCERI receptors and causing basophil degranulation. This process could conceivably lead to inflammation and tissue damage in BP skin. When taken overall, these findings provide strong evidence for an additional complement-independent, Th2-dependent, eosinophil-mediated pathway that contributes to tissue damage and clinical features in BP. A summary of IgE and eosinophil dependent pathomechanisms are shown in **Figure 2**.

Finally, investigation regarding IgM antibodies in pemphigoid also highlights the possibility of complement-independent mechanisms in these diseases. Cases of IgM bullous pemphigoid have been documented, with such patients exhibiting linear deposition of both IgM and C3 (120–125). In addition, several patients with only IgM deposition have been identified (126, 127). A recently published article from Boch et al. describes three patients with ‘non-bullous’ pemphigoid who presented with erythematous papules and plaques, two of whom displayed exclusive IgM deposition at the BMZ in the complete absence of complement. The other patient showed weak complement binding. Additionally all three patients demonstrated no serum complement activation capacity (128). Thus, complement-independent mechanisms may play a more prominent role in unique subtypes of AIBD such as this. However, as C3c deposition has been shown to be significantly decreased in non-bullous patients as compared to those with blisters (33, 55), these findings continue to reinforce the central importance of complement activation in actual blister formation as well.

## Lessons From the Bedside and Targeted Therapies

The presence of complement-dependent and independent mechanisms in BP not only highlight the disease’s biologic complexity, but treatment challenges. Currently, several inhibitors of complement components are under investigation for the treatment of BP. Other than identifying the presence of complement on the epidermal BMZ which has significant limitations, it remains a challenge to stratify patients who may have significant contribution from complement-independent pathways. Nonetheless, biomarkers of complement independent pathways can be utilized to target these pathways.

Patients with BP often exhibit elevated serum IgE levels and circulating BP180- and BP230-specific IgE autoantibodies. These findings provide support to the idea that IgE has a role in BP pathogenesis (129, 130). In fact, it is thought that IgE autoantibodies directed against the ectodomain of BP180 are first bound to FcεRI on mast cells and eosinophils. This binding subsequently promotes degranulation and initiates an inflammatory reaction resulting in further tissue damage and blister formation (44, 97, 111, 131–133). In addition, binding of specific IgE autoantibodies to the ectodomain of BP180 on basal keratinocytes also triggers internalization of BP180 (see above) and thereby contributes to cell-substrate disadhesion (44, 119, 134). As a result, a humanized mAb that inhibits IgE binding to its high-affinity receptor (FcεRI), omalizumab (an approved treatment for severe asthma and chronic spontaneous urticaria), represents a logical alternative drug for BP. In 2009 Fairley et al. first reported the beneficial effect of omalizumab in a BP patient poorly controlled by oral corticosteroids, azathioprine, and minocycline. After 16 weeks of treatment, several patients showed a significant improvement despite discontinuation of corticosteroids. Since this first report, several case series have confirmed the value of omalizumab as either monotherapy or adjuvant therapy in patients with various forms of BP (105, 135–138). We have also successfully used omalizumab in a number of BP patients and found that omalizumab treatment results in a sharp decrease of FcεRI expression on circulating basophils and a strong reduction of FcεRI+ cells in the skin of treated patients (129). Our results are thus in line with the idea that omalizumab is able to sequester free IgE and prevent its binding to its high-affinity IgE receptor, FcεRI (139–141). This process has been proposed to then downregulate the expression of FcεRI on mast cells and basophils as well as antigen-presenting cells (139).

In addition to omalizumab, there are other biological targeted therapies in development for BP (142). Dupilumab, a human IgG4 monoclonal antibody binding the IL4-Rα inhibits IL-4 and IL-13. It is approved in atopic dermatitis and is being studied in BP, and has several reports of treatment success (7, 143–145). While a phase 2 study of mepolizumab, a humanized IgG1 monoclonal antibody targeting IL-5, was unsuccessful in BP (146), benralizumab, a human IgG1 monoclonal antibody targeting IL5-Rα that leads to apoptosis of eosinophils and basophils, is being studied.

Together, these observations corroborate the idea that complement-independent mechanisms, which play a role in BP pathogenesis, offer additional therapeutic targets beneficial for affected patients. The recent, promising results of the complement inhibitor nomacopan continue to reflect the well-established role of complement-dependent mechanisms as the primary driver in the pathogenesis of BP (8). As such, downregulation of the complement pathway should remain the priority for investigation of therapeutic targets in this disease. However, given the evidence laid out above for the existence of complement-independent mechanisms, it is reasonable to conclude that specific targeting of these pathways may offer additional benefit to patients in the future, or be a major treatment option for a subset of patients in which these mechanisms are predominant.

## Conclusions

Increased insight into complement-independent mechanisms in BP has not only improved our understanding of BP pathogenesis but has also significant translational implication. The increasing knowledge gained from studies dissecting IgE- and eosinophil-dependent pathways have highlighted the importance of extending the therapeutic horizons beyond those predominantly focusing on complement-mediated pathways. Hence, several avenues remain to be therapeutically explored. For example, blocking the production of pro-inflammatory mediators released by keratinocytes represents a potential approach by which BP may be improved. Likewise, induction of BP180 expression in basal keratinocytes to compensate for BP-IgG induced BP180 loss could also have a beneficial effect.

The extent to which complement-dependent or -independent mechanisms contribute to phenotypic presentation remains to be determined. Dissecting contributory pathways has significant impact on personalized treatments. For example, autoantibodies to non-NC16a epitopes have been associated with a pauci-inflammatory phenotype (147). Could this be sufficiently explained by the lack of NC16a-mediated endocytosis and subsequent expression of pro-inflammatory molecules? Several other questions remain. Are anti-NC16a induced keratinocyte pro-inflammatory molecules sufficient to induce granulocyte infiltration in the absence of complement? Does anti-BP180 depletion on keratinocytes lead to skin fragility in patients or is this primarily protease driven? Do keratinocytes express other pro-inflammatory cytokines/chemokines that may account for the eosinophilia typically seen in BP? Is keratinocyte derived IL-8 the major inducer of neutrophil chemotaxis or complement? Can the presence or absence of complement fixing antibodies predict responses to different therapies?

While the whole-body application of topical corticosteroids may affect these pathways, their systemic absorption, local side effects, and their practical use in elderly patients constitute a therapeutic hurdle (148, 149). It is likely that in the near future, the possibility to more rapidly and easily obtain a comprehensive characterization of complement-independent pathways activated in lesional tissues obtained from BP patients, for example using gene expression profiling, transcriptomics and proteomics, will provide a means to better tailor the therapy plan to the individual affected by BP.

## Author Contributions

All authors contributed to the writing and review of the manuscript. All authors approved of the final manuscript.

## Funding

KA is supported in part by Office of Research Infrastructure Programs of the National Institute of Health (R21OD030057).

## Conflict of Interest

KA has served as a consultant for AstraZeneca, Akari Therapeutics, Argenx, as well as received research support from AstraZeneca.

The remaining authors declare that the research was conducted in the absence of any commercial or financial relationships that could be construed as a potential conflict of interest.

## Publisher’s Note

All claims expressed in this article are solely those of the authors and do not necessarily represent those of their affiliated organizations, or those of the publisher, the editors and the reviewers. Any product that may be evaluated in this article, or claim that may be made by its manufacturer, is not guaranteed or endorsed by the publisher.
